# Bluejay 1.0: genome browsing and comparison with rich customization provision and dynamic resource linking

**DOI:** 10.1186/1471-2105-9-450

**Published:** 2008-10-22

**Authors:** Jung Soh, Paul MK Gordon, Morgan L Taschuk, Anguo Dong, Andrew C Ah-Seng, Andrei L Turinsky, Christoph W Sensen

**Affiliations:** 1 University of Calgary, Faculty of Medicine, Sun Center of Excellence for Visual Genomics, 3330 Hospital Drive NW, Calgary, AB, T2N 4N1, Canada; 2 Centre for Computational Biology, Hospital for Sick Children, 555 University Avenue, Toronto, ON, M5G 1X8, Canada

## Abstract

**Background:**

The Bluejay genome browser has been developed over several years to address the challenges posed by the ever increasing number of data types as well as the increasing volume of data in genome research. Beginning with a browser capable of rendering views of XML-based genomic information and providing scalable vector graphics output, we have now completed version 1.0 of the system with many additional features. Our development efforts were guided by our observation that biologists who use both gene expression profiling and comparative genomics gain functional insights above and beyond those provided by traditional per-gene analyses.

**Results:**

Bluejay 1.0 is a genome viewer integrating genome annotation with: (i) gene expression information; and (ii) comparative analysis with an unlimited number of other genomes in the same view. This allows the biologist to see a gene not just in the context of its genome, but also its regulation and its evolution. Bluejay now has rich provision for personalization by users: (i) numerous display customization features; (ii) the availability of waypoints for marking multiple points of interest on a genome and subsequently utilizing them; and (iii) the ability to take user relevance feedback of annotated genes or textual items to offer personalized recommendations. Bluejay 1.0 also embeds the Seahawk browser for the Moby protocol, enabling users to seamlessly invoke hundreds of Web Services on genomic data of interest without any hard-coding.

**Conclusion:**

Bluejay offers a unique set of customizable genome-browsing features, with the goal of allowing biologists to quickly focus on, analyze, compare, and retrieve related information on the parts of the genomic data they are most interested in. We expect these capabilities of Bluejay to benefit the many biologists who want to answer complex questions using the information available from completely sequenced genomes.

## Background

### Pre-1.0 Bluejay

The features of Bluejay (Browser for Linear Units in Java) prior to the release of version 1.0 were described in [1,2]. We briefly summarize them as background information, which is necessary to put the new features of Bluejay 1.0, which have not been described elsewhere, into context.

Bluejay supports Moby [3], a protocol consisting of a common XML object ontology for biological entities and a standardized request/response mechanism for automating Web-based analysis. This allows the user to seamlessly link from the visualized data, internally represented as a Document Object Model (DOM) [4], to Moby-compliant Web services by embedding the Seahawk Moby client [5]. The TIGR MultiExperiment Viewer (MeV) [6] is integrated into Bluejay such that it functions as a fully embedded module (see Additional file 1). Through this integration, Bluejay can show gene expression values in a genomic context, enabling biologists to draw additional inferences from gene expression analyses (e.g., operon structures). Bluejay loads URL-based data via a proxy Web Service. If an XML file with a large amount of data is requested, the proxy performs data skeletonization on-the-fly, using an eXtensible Stylesheet Language Transformation (XSLT) [7], which allows Bluejay to run on a memory-limited computing environment (and as an applet).

### Novel features of Bluejay 1.0

Bluejay is a unique genome browser that offers numerous features not available in other genome browsers. Bluejay 1.0 adds comparative genomics and enriched customization/personalization capabilities in order to provide the biologist with an elegant way to simultaneously view genes in multiple contexts (genomic, comparative and expression). The new features include: (i) visual comparison of multiple genomes on a single canvas; (ii) genomic information recommendation reflecting user preference; and (iii) flagging elements of a genome and utilizing the flags for navigation and comparison.

## Implementation

Bluejay is available from the project home page [8] as an applet, Java Web Start, or a standalone application. Bluejay is implemented in Java 1.5 and uses a number of open source libraries from the Apache Foundation [9]. The details on how the libraries are integrated into Bluejay are described in [2]. Here we focus on how a biologist can use Bluejay's genomic, comparative and expression contexts to enhance their understanding of a gene or set of genes of interest.

**Figure 1:**
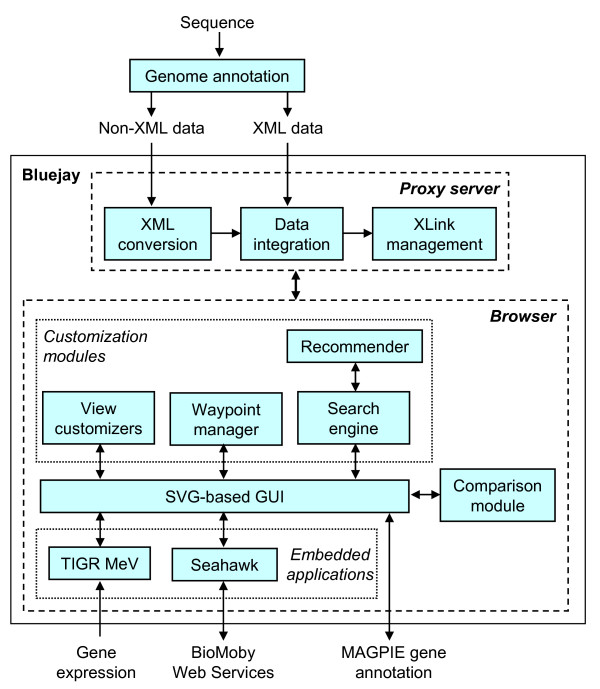
**Conceptual structure of Bluejay 1.0**. Bluejay can load XML and non-XML genome annotation data, which are internally represented as an XML document, with XLinks inserted. The user interacts with Bluejay through the browser GUI, which communicates with the comparison module, customization modules, embedded applications, and gene annotation pages.

Figure 1 illustrates the high-level architecture of Bluejay 1.0. Genome sequence data first needs to be analyzed using an annotation tool (e.g., MAGPIE [10]) in order to obtain meaningful visualization in Bluejay. Alternatively, annotated genome sequences available from public repositories (e.g., GenBank) can also be loaded into Bluejay. In either case, the resulting annotated sequence is loaded into Bluejay either in XML (the default format) or non-XML format; by requesting a URL or opening a local file. Bluejay comes with built-in bookmarks to load public genomes available from the MAGPIE home page [11]. Any non-XML data are converted internally to Bioseq-set XML documents. Bluejay supports the following XML dialects for genome annotation:

• AGAVE: Architecture for Genomic Annotation, Visualization and Exchange. 

• TIGR: The format in which TIGR (The Institute for Genomic Research) annotations are distributed. 

• Bioseq-set: The output format for XML option in readseq, a popular sequence format conversion program. 

• NCBI_GBSeq: NCBI GenBank's XML sequence data format. 

The XLink standard [12] is used to hyperlink visual representations of genomic features to external data. For example, MAGPIE gene annotation pages (if available) can be launched by clicking on a gene. Custom XML documents created by users can include XLinks to any Web page. XLinks are inserted before the annotated sequence is internally represented as a DOM tree. The graphics engine uses Scalable Vector Graphics (SVG) [12] to render the DOM tree. This supports almost unlimited semantic zooming and publication-quality image output. Through the browser GUI, users can customize their viewing preferences, set waypoints for easier navigation, and invoke the search engine for personalized information retrieval. The comparison module is automatically entered and exited depending on the number of genome sequences loaded.

Two standalone software applications are currently embedded in Bluejay for linking to external resources accessible through the browser GUI: Seahawk for accessing Moby-compliant Web Services and TIGR MeV for gene expression analysis. Currently, Seahawk is incorporated into Bluejay as a single Java Archive (JAR) file. No specially designed application programming interface (API) is required to use the functionality of Seahawk from within Bluejay [5]. This approach also makes it easier to update Seahawk when a new release becomes available, avoiding the need to change the Bluejay source code. TIGR MeV has been integrated at the source level, because we needed to extend its functionality to display gene expression values within the context of the corresponding genomes.

## Results

### Whole genome comparison

Bluejay is capable of visualizing multiple genomes in a single display for intuitive comparison. The comparison mode is automatically activated if the user loads more than one genome, and exited when only one genome remains loaded. A central feature for genome comparison is the display of lines that link common genes based on their gene classification. This helps the user to instantly estimate how functionally similar the compared genomes are. For example, Bluejay comes with a bookmark that lets the user access genome data annotated using the MAGPIE system, which uses Gene Ontology (GO) [14] as the gene classification system. As GO allows standardized multi-level hierarchical classification of a gene, each gene is linked only to the physically closest gene in another genome with the same classification at the most detailed GO level, if such a gene exists. However, it should be noted that GO is not the only gene classification system that can be used in Bluejay to compare genes. As the textual gene descriptions are compared to match genes, any kind of gene classification system that the user wants to use can be accommodated in Bluejay.

In the comparison mode of Bluejay, all genome displays are scaled with respect to the length of the first genome for ease of visual comparison. This means that all circular genomes are displayed as a complete circle and the all linear genome displays are normalized to the length of the first genome. Thus, for circular representation, the distance between two genes used for finding the nearest genes is in units of the angle difference between the two gene locations.

An enhanced feature related to gene linking is the automatic gene-level alignment of circular genomes to minimize the sum of the angular distances for all linked pairs of genes. This amounts to minimization of linking distances to find the best global alignment of closely related genes. When this feature is enabled, the outer sequence is automatically rotated to the best-aligned position. The user can then see the functional similarity of the genes in the two sequences, with the effect of base position differences minimized.

**Figure 2:**
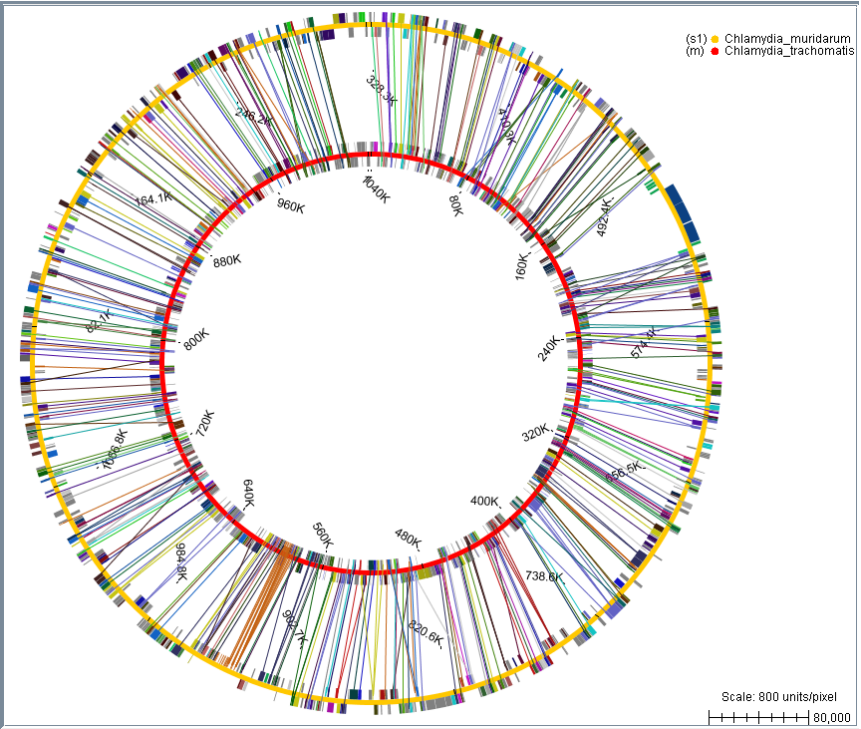
**Bacterial genome comparison**. The comparison of two bacterial genomes (*Chlamydia trachomatis *and *Chlamydia muridarum*) shows that they share many genes, which belong to the same Gene Ontology (GO) classification, as indicated by the linking lines. Only those links with an angular distance less than 2% of 360 degrees are shown.

Another useful feature of Bluejay for comparing genomes is the ability to selectively show or hide links between genes. The selection is based on limiting the linking distance as a percentage of the length of the first genome (for linear genomes) or a percentage of 360 degrees (for circular genomes), adjustable by the user. This allows the user to visually perceive the degree of similarity of the compared genomes, based on the positions of similar genes. Figure 2 shows an example of comparing two *Chlamydia *genus genomes using the aforementioned comparison features [15] (for more details of the comparison steps, see Additional file 2).

**Figure 3:**
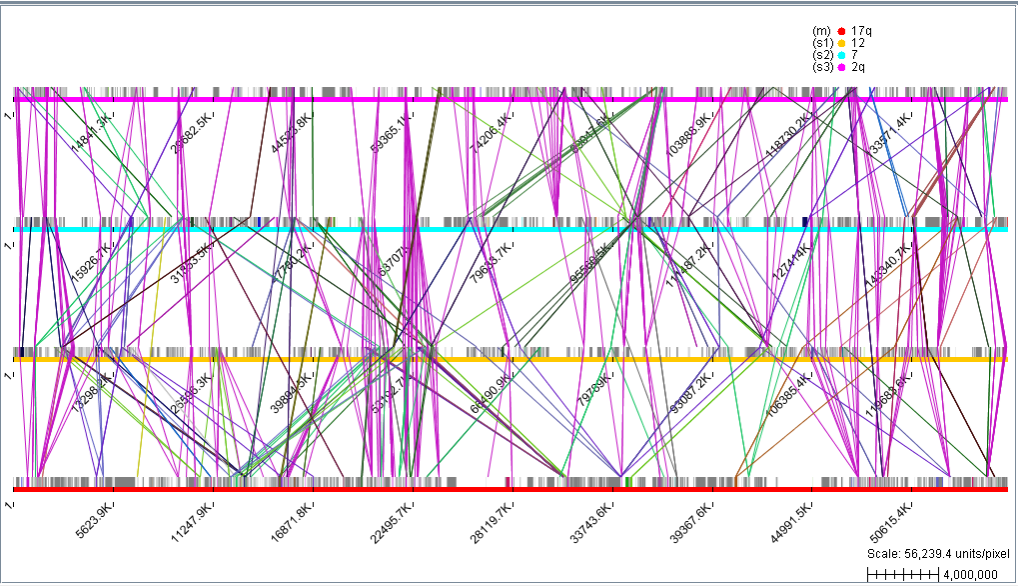
**Human chromosome comparison**. The comparison of human chromosomes 17q, 12, 7, and 2q shows that many gene families, such as homeobox (HOX) and epidermal growth factor receptor (ErbB) genes, are duplicated on several chromosomes.

Another example of using the comparative genomics functionality of Bluejay is shown in Figure 3, where four human chromosomes (or chromosomal arms) are being compared. It has been known that the human chromosomes contain many instances of gene family duplications [15]. Using Bluejay, we can visually confirm that a selected subset of human chromosomes indeed has a number of duplicated genes. The direction of gene linking is by default from the first genome to other genomes (as in Figure 2), but this direction can be reversed, or even both directional links can be shown (as in Figure 3).

Bluejay supports the manual operation on genomes loaded for comparison, mainly because there may be a need to compare specific genes that are not proximate in the automatic global genome alignment. All loaded circular sequences may be rotated together or individually. The lines linking common genes are dynamically repositioned when the user rotates or unloads a sequence. Finding out the correct rotation angle for a sequence to align genes used to be a trial-and-error process. We have now automated the rotation operation to align a set of genes by taking advantage of the waypoints capability (described later in detail).

### Personalized search via a hybrid recommender system

When viewing a large amount of annotated information at once in a genome browser, the user is easily overwhelmed by the quantity of data. Even if the user is familiar with the data itself, thousands of genes, oligonucleotides, gene expression values, and their annotations may be displayed simultaneously, making it difficult to locate the desired information. This problem is compounded when multiple genomes are being viewed simultaneously. Even a search engine may not be flexible enough, because a single gene or function may be listed under several synonyms. A recommender system that helps users to locate specific, relevant, personalized information in complex and heterogeneous genomic datasets was therefore implemented.

**Figure 4:**
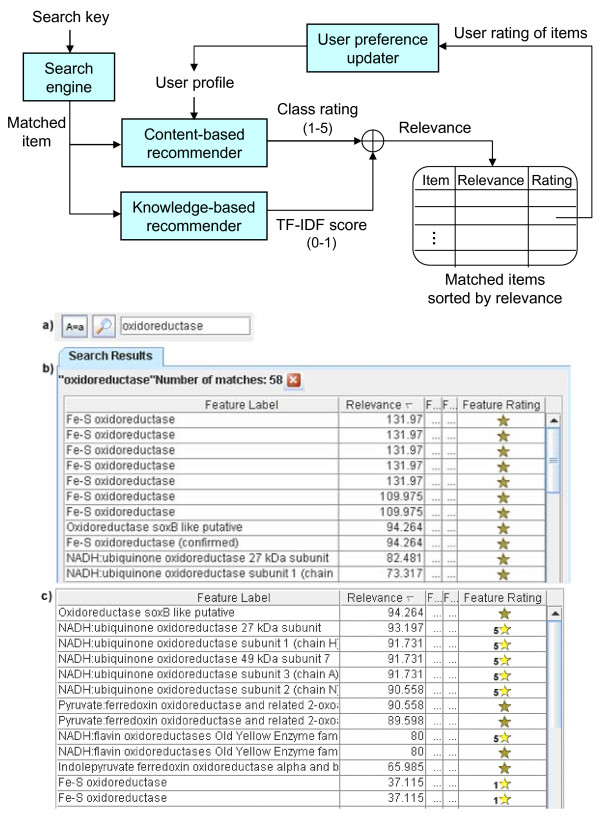
**Hybrid recommender and personalized search**. A matched item from the search engine is ranked, based on previously learned user preferences, and then refined by the system's knowledge of its importance. An example: (a) A search for "oxidoreductase"' is performed on the *Sulfolobus solfataricus *genome; (b) The relevance for each search item is listed in the Relevance column. Items containing "Fe-S" have the highest relevance due to TF-IDF ranking. The user subsequently rates items containing Fe-S low (1) and items containing NADH high (5) using the Feature Rating column (rating action not shown); (c) In a subsequent search for oxidoreductase, the items containing NADH are rated above those containing Fe-S, based on the user profile.

The Bluejay system includes a hybrid recommender system composed of a knowledge-based recommender and a content-based recommender [17], as part of its search engine (Figure 4). The knowledge-based component is implemented using the term frequency-inverse document frequency (TF-IDF) text mining algorithm [18] as follows: (i) matched items, annotated genes and other textual items in Bluejay's search results are regarded as "documents"; and (ii) single words found in each document in the search results are taken as "terms". When there is no user profile, the algorithm solely determines the ranking of the search results. Since gene annotations vary widely in length, normalization is applied to the rankings to lessen the influence of document sizes.

Because of the brevity of genomic annotations in comparison to typical documents in text mining, the TF-IDF algorithm alone is not sufficient to determine the ranking of search results. The content-based component uses a previously learned user profile to reflect the user preference of documents. A user creates a personal profile by rating matched items on a 5-point Likert scale [19], with 1 being "not useful" and 5 being "very useful". As an item is rated, the relevance values of all keywords that comprise it (i.e., "terms" in a "document") are continuously updated in the user's profile, using Rocchio's algorithm [20]. The user profile consists of the average vectors of each "usefulness" class, corresponding to the Likert scale. Once the user profile is created, it can help to classify new matched items. The distances between the learned user preference vectors and the new item are calculated using the cosine similarity measure, which determines the class (1 to 5). The TF-IDF score (0 to 1) is added to refine the order of items within a class, resulting in a relevance score represented as a percentage.

The recommender system is invoked via the "Search Results" window. Updated in response to user interactions, the window displays a sorted table of search results that are deemed most relevant to the user's needs. Users may change their usefulness rating of any matched item by choosing one of five predefined Likert scores from the pull-down menu in the "Feature Rating" column. Figure 4 illustrates how the recommendation order based on TF-IDF alone is altered after the user rates the items.

### Waypoints for customized navigation and comparison

Biologists are often interested in viewing only a small portion of a genome. Navigating within a large genome usually entails scrolling until the desired part is in view or typing in a base number to see the view around it. Using these methods, the user has to spend considerable time to focus on the desired part of the genome.

**Figure 5:**
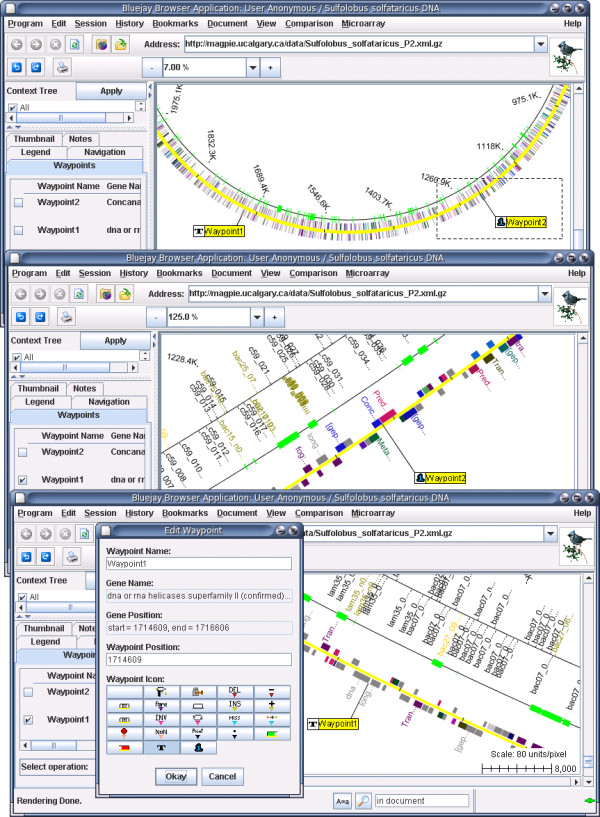
**Customized navigation using waypoints**. The user sets two waypoints and zooms in the area around *Waypoint2 *(top). The selected area is displayed in greater detail, then the user clicks *Waypoint1 *in the "Waypoints" tab (middle). The focus changes to *Waypoint1*, then the user selects "Edit Waypoint" action (bottom) to change the attributes.

With Bluejay, we now offer a significantly more intuitive method for exploring large genomes by using the notion of a waypoint, which is loosely defined as a coordinate that identifies a point in 2D or 3D space. In Bluejay, waypoints are represented as a collection of flags with positional and descriptive information to mark points of interest on a genome. The user can set a waypoint within the sequence to highlight a specific gene or sequence feature (such as promoters or terminators). Figure 5 shows a typical use of waypoints to quickly focus on two regions of interest. Once a waypoint is set, a number of operations can be performed, including focusing on it or aligning several genomes at a waypoint with the same name within multiple genomes.

Viewing a genome at the nucleotide text level is possible in Bluejay. In a text mode, a waypoint can be set not only with respect to a whole gene but also at a base position, helping the user to investigate bases as well as whole genes. For example, in the "horizontal sequential" text mode, a genome is displayed as an elongated string of characters (for an example of setting multiple waypoints in the text mode, see Additional file 3).

The usage of waypoints in combination with genome rotation for the comparison of circular genomes adds to the utility of waypoints. There is often the need to align multiple circular genomes at specific genes to investigate how similar they are around those genes. Without waypoints, the user would try rotating the genomes by some angles until the genes of interest align more or less. Waypoints in Bluejay provide the user with the ability to flag multiple genes with the same name and align them at those flags, without estimating how much rotation might be necessary for the exact alignment (for an example of aligning multiple genomes at a gene of interest, see Additional file 4).

## Discussion

### Genome comparison

Bluejay offers the ability to display multiple genomes in the same visual display for direct comparison, along with lines linking genes of the same classification, which immediately shows the degree of similarity of the genomes. Automatic alignment of genes based on linking distance minimization and user-directed waypoint-based alignments of genes are novel features for genome comparison. For example, the UCSC Genome Browser [27] can display multiple genomes in different tracks to show base-level alignment and allows the user to sort genes based on several different gene information categories. However, it does not let the user visually align genomes at user-specified genes. However, there is no provision for the user to tag specific genes and use them as anchors for visually aligning genomes. The Ensembl Genome Browser [24] provides a set of comparative genomics tools based on multiple sequence alignment, but visually comparing the sequences at the gene level is not possible.

The gene-level comparison of genomes in Bluejay adds to the existing capability of sequence match-based comparison approaches such as the Blast programs [21], which are widely used for searching protein and nucleotide databases to identify sequence similarities. MUMmer [22] is a fast comparison tool to align two large nucleotide sequences. These tools are essentially search or alignment tools that do not provide the user with the information on the similarity of gene functions. A more recent genome comparison tool called M-GCAT [23] can generate lines to link maximal unique matches occurring in multiple sequences. This tool displays sequence match results to reveal similarities of genomes at the sequence level, whereas Bluejay visualizes annotated functional descriptions of genes which allows users to compare genomes at the gene level.

Any gene classification system can be adopted for genome comparison in Bluejay. For example, in particular analysis projects, we have successfully replaced GO terms with Ensembl [24] paralog family labels for classification of genes in the source XML files. As GO provides a standardized classification scheme that allows multiple levels of classification for a single gene, adopting GO classifications of genes allows users to compare genomes based on gene functions. However, the current approach is clearly limited in detecting genome rearrangements, for which other comparison approaches that depend on sequence search and alignment are better suited. For example, four genomes within the bacteria *Neisseria *genus were compared using Blastn [21] which identified four different types of genome polymorphisms including a genome-wide inversion [25]. Since any object displayed in Bluejay, including the genes, act as hyperlinks to additional gene-specific information through the MAGPIE annotation pages [11] or BioMoby-compatible Web services [3,5], using these additional sources of data for comparing genomes would be a useful future addition to the current comparison functionality of Bluejay.

While Bluejay is primarily intended for comparing complete genomes, it is possible to display incomplete genomes or ESTs, but this requires a reference genome onto which sequence fragments are placed in order to create a pseudogenome. In this mode, the locations of sequence fragments must be verified by performing sequence alignment ahead of time, as otherwise displaying fragments along an imaginary backbone could be misleading.

### Customization for exploring genomic information

Bluejay is the first genome browser we know of with a rich set of waypoint management functions that enables the user to easily navigate within a genome. We have found that in order to visualize the region of interest in a genome, a method of marking positions of a sequence and performing operations on the user-defined markers is far more intuitive than scrolling around or typing in base numbers. An innovative, additional use of waypoints for user-directed gene alignment for comparison adds to the usability of waypoints. To our knowledge this conceptually simple but functionally powerful feature has not been exploited for genome exploration in any other system.

Bluejay's recommender system helps bridge the gap between the vastness of genomic data and the specificity of users' needs. Especially in genomes with large numbers of annotations, targeting search results using semantic knowledge about the user's preferences can help the user to be productive. To our knowledge, no other genome browser has provided a recommender system yet. GBrowse [26] and the UCSC Genome Browser [27] have search engines and the capability for user profiles, but neither recommends information based on learned user preferences. The Ensembl genome annotation source [24] performs some data mining by searching multiple databases, but it does not record the user's interests.

### Dynamic resource discovery and linking

Bluejay provides interoperability and extensibility by dynamically finding and linking external data and biological Web services via standard protocols. Most popular genome browsers other than Bluejay have limited capabilities of external data and services linking. The UCSC Genome Browser does not provide the user with the option of directly linking out to external biological resources [27]. Ensembl can link the distributed annotation system (DAS) resource [28] and contains hardcoded hyperlinks that link to external services. The NCBI Map Viewer [29] provides a "LinkOut" link to access information about a gene. Most of the links in the other browsers are hardcoded, which creates maintenance issues for the tool creators.

The visualization of gene expression levels in the context of a whole genome in Bluejay enables biologists to gain valuable insights on gene functions. For smaller genomes, individual genes and their expression values can be investigated without any special visualization methods. However, larger genomes with thousands of genes (e.g., the *Sulfolobus solfataricus *genome with almost 3000 genes) require viewing the genome and expression values as a whole. Bluejay has been successfully used by biologists for a number of microarray studies [30,31], which demonstrates its usability. To date, no other genome browser we know of provides this integrative visualization functionality. We expect Bluejay to be useful to many researchers who can take advantage of the combined genomic, transcriptional and comparative contexts to more easily answer biological questions.

## Conclusion

Bluejay version 1.0 is one of the more comprehensive visual environments for exploring genomes and related biological data. In addition to visualizing the data for a single genome, Bluejay can now visualize multiple whole genomes and provides the user with a set of operations useful for genome comparison. The user can use waypoints for navigating within a genome and for comparing genomes. The hybrid information recommender system coupled with the search engine provides personalized genomic information. Dynamic discovery and linking of Moby Web Services from Bluejay enables the user to seamlessly connect to diverse biological resources while keeping the application itself relatively lightweight. Bluejay also provides access to a comprehensive package (TIGR MeV) for the analysis of microarray data, with the additional capability of graphical display of gene expression values together with genomic data. We believe that these unique capabilities represent an essential collection of visually oriented tools for bioinformatics analysis in general, and genome/transcriptome analysis in particular.

## Availability and requirements

**Project name: **Bluejay

**Project home page: **

**Operating systems: **Platform independent

**Programming language: **Java 1.5 or higher

**License: **GNU Lesser General Public License (LGPL)

**Any restrictions to use by non-academics: **None

## Authors' contributions

JS improved the waypoint manager, the comparison module, and the text view modes, and integrated them into Bluejay 1.0. PMG designed the software architecture of Bluejay and developed most of its core classes. MLT coded earlier versions of the waypoint manager and the text view modes, and also implemented the recommender system. AD coded an earlier version of the genome comparison module. ACA integrated TIGR MeV into Bluejay and coded the gene expression visualization module. ALT developed most of the SVG graphics engine. CWS was responsible for scientific guidance on all of the presented aspects of Bluejay. JS wrote the manuscript with contributions by PMG, and critical review by CWS. All authors read and approved the final manuscript.

## Supplementary Material

Additional file 1 **Interaction between Bluejay and embedded TIGR MeV**. Microarray data parsing and cluster analysis are handled by TIGR MeV. The expression/cluster data are integrated with the genome data to produce a coherent visual representation. Tables of expression values and images of the genome along with visualized expression values can then be exported. Click here for file

Additional file 2 **Bacterial genome comparison**. The comparison of two bacterial genomes shows that they share many genes with the same Gene Ontology (GO) classification, as indicated by the linking lines: (i) *Chlamydia trachomatis *and *Chlamydia muridarum *genomes are compared (top); (ii) the two genomes are automatically aligned to have the minimum possible total angular linking distance (bottom). Click here for file

Additional file 3 **Using waypoints in a text mode**. In the text mode, the genes are shown as coloured rectangles over the bases and waypoints can be set at individual base positions. In this case, the possible location of a frameshift is seen at the nucleotide level, with the annotated ORF boundaries as waypoints. Click here for file

Additional file 4 **Aligning genomes by waypoints**. A waypoint named dpoII is set at the appropriate location in each of three *Sulfolobus *spp. genomes, and the "Align at Waypoint" action is selected to align the genomes at the dpoII gene by appropriately rotating the outer genomes. This makes visual comparison of the gene's structure in all three species easier. Click here for file
