# Supplementary figures and images for: Bluejay 1.0: genome browsing and comparison with rich customization provision and dynamic resource linking
Source: BMC Bioinformatics. 2008 Oct 22;9:450. doi: 10.1186/1471-2105-9-450 (PMC2590619; doi:10.1186/1471-2105-9-450)

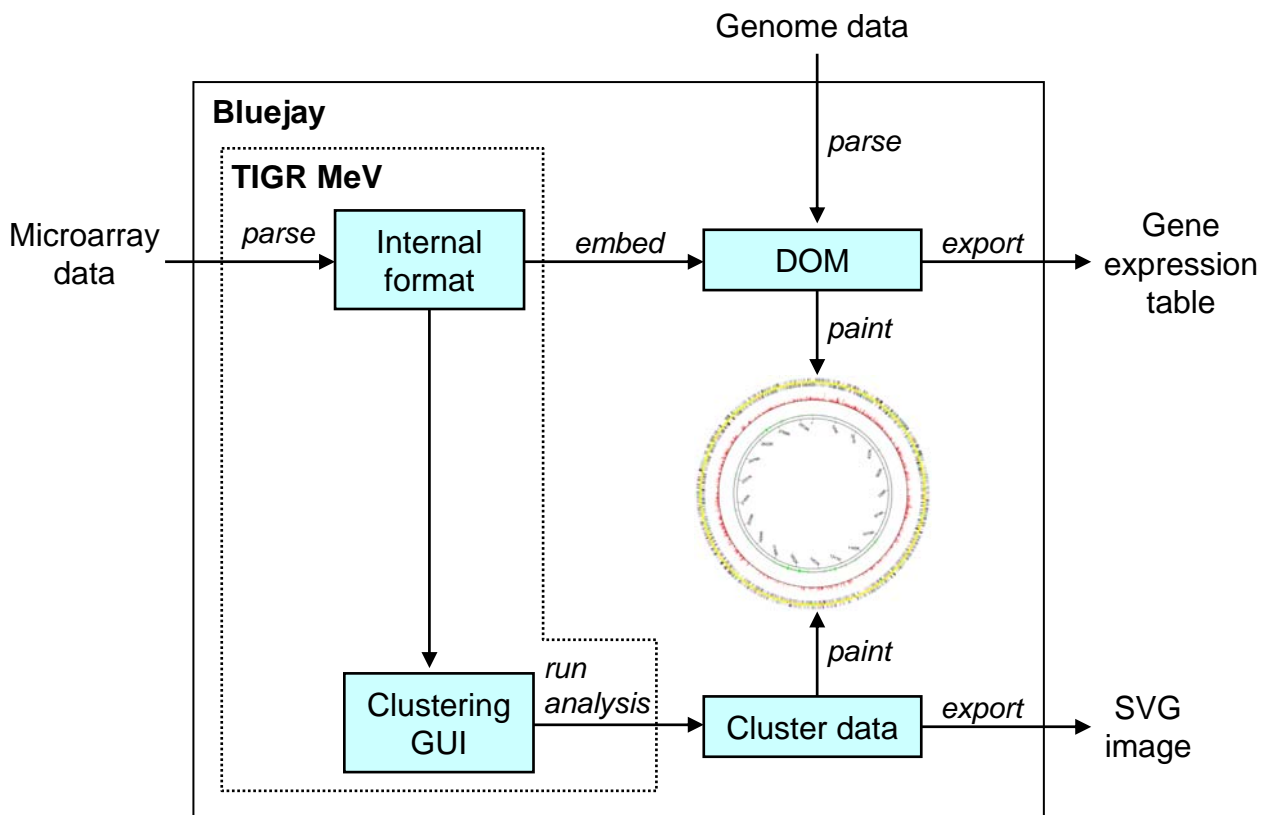

Supplement: Additional file 1 — Interaction between Bluejay and embedded TIGR MeV. Microarray data parsing and cluster analysis are handled by TIGR MeV. The expression/cluster data are integrated with the genome data to produce a coherent visual representation. Tables of expression values and images of the genome along with visualized expression values can then be exported. [file 1471-2105-9-450-S1.pdf]

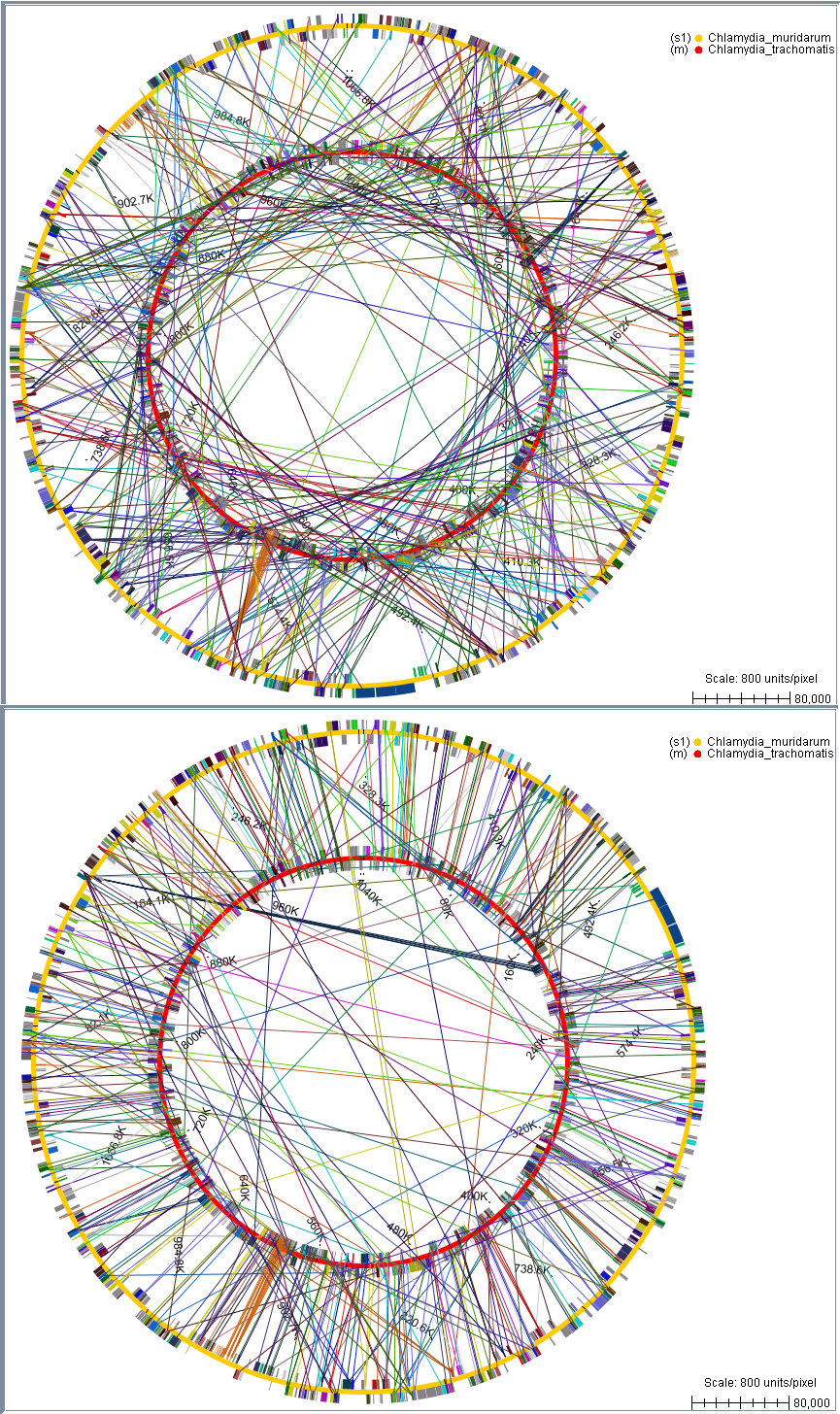

Supplement: Additional file 2 — Bacterial genome comparison. The comparison of two bacterial genomes shows that they share many genes with the same Gene Ontology (GO) classification, as indicated by the linking lines: (i) Chlamydia trachomatis and Chlamydia muridarum genomes are compared (top); (ii) the two genomes are automatically aligned to have the minimum possible total angular linking distance (bottom). [file 1471-2105-9-450-S2.png]

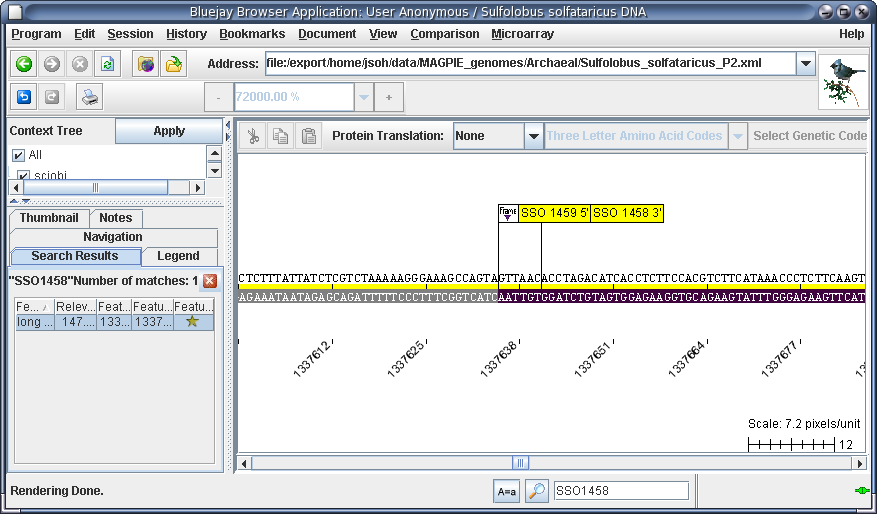

Supplement: Additional file 3 — Using waypoints in a text mode. In the text mode, the genes are shown as coloured rectangles over the bases and waypoints can be set at individual base positions. In this case, the possible location of a frameshift is seen at the nucleotide level, with the annotated ORF boundaries as waypoints. [file 1471-2105-9-450-S3.png]

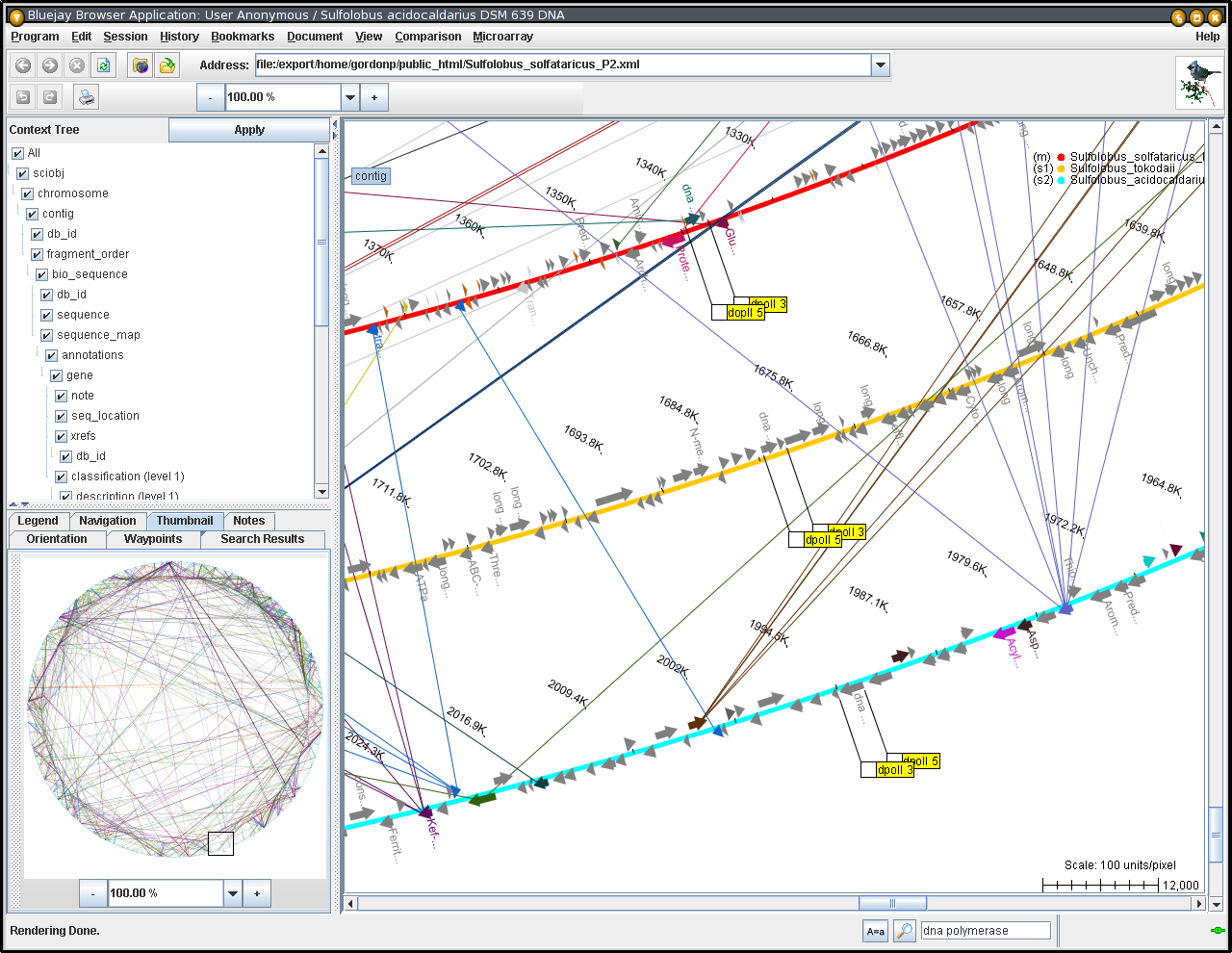

Supplement: Additional file 4 — Aligning genomes by waypoints. A waypoint named dpoII is set at the appropriate location in each of three Sulfolobus spp. genomes, and the "Align at Waypoint" action is selected to align the genomes at the dpoII gene by appropriately rotating the outer genomes. This makes visual comparison of the gene's structure in all three species easier. [file 1471-2105-9-450-S4.png]
